# Analysis of clinical characteristics of 617 patients with benign airway stenosis

**DOI:** 10.3389/fmed.2023.1202309

**Published:** 2023-07-20

**Authors:** Jinmei Wei, Shujuan Qin, Wentao Li, Yan Chen, Tingmei Feng, Yuhui Wei, Sen Tan, Guangnan Liu

**Affiliations:** ^1^Department of Respiratory and Critical Medicine, The Second Affiliated Hospital of Guangxi Medical University, Nanning, China; ^2^Guangxi Medical University, Nanning, China; ^3^Department of Pulmonary and Critical Care Medicine, Guigang City People's Hospital, Guigang, China

**Keywords:** benign airway stenosis, etiology, tuberculous tracheobronchial stenosis, traumatic airway stenosis, airway foreign body, epithelial-mesenchymal transition, fibrosis

## Abstract

**Introduction:**

Benign airway stenosis (BAS), namely airway narrowing caused by a variety of benign lesions, can lead to varying degrees of breathing difficulties and even death due to asphyxia. This study aimed to elucidate the clinical characteristics of BAS, including etiology, treatment and pathology, by analyzing the clinical data of BAS patients.

**Methods:**

A retrospective analysis was conducted using the clinical data of 617 BAS cases from January 2017 to December 2022. The pathological characteristics of the tissues were assessed by hematoxylin–eosin (H&E) and Masson’s staining. Besides, protein expression levels were determined by immunohistochemistry (IHC).

**Results:**

A total of 617 patients were included (333 females [53.97%] and 284 males [46.03%]), with an average age of 48.93 ± 18.30 (range 14–87). Tuberculosis (*n* = 306, 49.59%) and trauma (*n* = 179, 29.02%) were the two leading etiologies of BAS, followed by airway foreign bodies (FB, *n* = 74, 11.99%), external compression (*n* = 25, 4.05%) and other etiologies (*n* = 33, 5.35%). Among 306 tuberculous tracheobronchial stenosis (TBTS) cases, most were females (*n* = 215, 70.26%), and TBTS mainly occurred in the left main bronchus (*n* = 97, 31.70%), followed by the right middle bronchus (*n* = 70 cases, 22.88%). The majority of TBTS patients (*n* = 259, 84.64%) were treated by interventional therapy. The condition of 179 BAS patients was ascribed to trauma, such as tracheal intubation (*n* = 92, 51.40%), tracheotomy (*n* = 69, 38.56%), injury (*n* = 15, 8.38%) and surgery (*n* = 3, 1.68%), which mostly took place in the trachea (*n* = 173, 96.65%). TAS patients mainly received interventional therapy (*n* = 168, 93.85%) and stent implantation (*n* = 47, 26.26%). The granulation tissues of BAS primarily featured inflammation, proliferation and fibrosis. IHC indicated the up-regulated expressions of transforming growth factor-β1 (TGF-β1), α-smooth muscle actin (α-SMA), collagen type I protein (COL-I) and vimentin, and the down-regulated expression of E-cadherin, which indicated fibrosis and epithelial-mesenchymal transition (EMT).

**Conclusion:**

Tuberculosis was the main etiology, and trauma was the secondary etiology. The granulation tissues of BAS were characterized by inflammation, fibrosis and probably EMT. Comprehensive interventional therapy is an effective method of treating BAS.

## 1. Introduction

Benign airway stenosis (BAS) refers to airway narrowing caused by various benign lesions, which can result in varying degrees of breathing difficulties and even death due to asphyxia (1). At present, its mainstream treatment methods include surgery and interventional and drug therapies (2). The therapeutical painful and subsequent complications in surgery or interventional therapy not only bring physical pain to patients but also cause psychological trauma to them and affect their quality of life (3, 4). It is necessary to adopt effective measures to prevent the aggravation of airway injury and pathological repair at the early stage, which is the fundamental prevention and treatment of BAS.

In this study, a retrospective analysis was conducted using the clinical data of BAS patients. The pathological characteristics of the tissues were evaluated by hematoxylin–eosin (H&E) and Masson’s staining methods. Besides, protein expression levels were detected by immunohistochemistry (IHC) to reveal the change of cytokines and proteins in BAS development to provide a basis for the clinical diagnosis and treatment of BAS.

## 2. Materials and methods

### 2.1. Case selection

BAS patients in the Second Affiliated Hospital of Guangxi Medical University in Guangxi, China were retrospectively analyzed from January 2017 to December 2022. Data concerning etiology, stenosis site, symptoms and treatments were collected. Selection criteria were as follows: patients who were aged over 14 and confirmed by bronchoscopy, chest computed tomography (CT) or pathology owing to various factors, such as endometrial tuberculosis, endotracheal intubation, tracheotomy, pulmonary surgery, external compression and airway foreign body (FB). Exclusion criteria were as follows: patients who were aged under 14, with unknown etiology unknown and insufficient data.

### 2.2. Pathological analysis

BAS granulations were obtained with bronchoscopy, and normal tissues were obtained from the surgical lobectomy of lung cancer. The specimens were fixed in a 4% paraformaldehyde solution for 48 h, then embedded and sliced for subsequent detection. Adjacent slices received H&E and Masson’s staining, and slides were scanned with an inverted microscope (Olympus, Japan). The primary antibodies for IHC, transforming growth factor-β1 (TGF-β1), E-cadherin, α-smooth muscle actin (α-SMA), collagen type I protein (COL-I) and vimentin for IHC were purchased from Servicebio, China. Slices for IHC were stained with TGF-β1, E-cadherin, α-SMA, COL-I and vimentin, then scanned with 3DHistech Digital Pathology System (Bio-One, China). Representative slice areas were shown in screenshots. Subsequently, Image J was used to read and analyze the positivity intensity of the measured areas.

### 2.3. Ethical statement

The study was approved by the Ethics Committee of the Second Affiliated Hospital of Guangxi Medical University, Nanning, China. Approval number [No. 2021-KY(0172)] and was conducted according to the principles outlined in the Declaration of Helsinki. Informed consent was obtained from all the subjects.

### 2.4. Statistical analysis

Statistical Package for the Social Sciences (SPSS) statistics software (version 26) was utilized to conduct all statistical analyses. GraphPad Prism 8 software was used for plotting. Qualitative variables were expressed as percent distributions in each category, and quantitative ones were expressed as means ± standard deviations and medians (ranges) for normally and non-normally distributed variables, respectively. The statistical test was an unpaired *t*-test, and a *p <* 0.05 was considered to show statistical significance.

## 3. Results

### 3.1. Demographic characteristics of patients

Among 881 airway stenosis cases included, 46 had unknown etiology, and 218 were diagnosed as malignant airway stenosis. As a result, 617 BAS patients were recruited. Of 218 malignant airway stenosis cases, most (*n* = 191, 87.61%) were attributed to lung cancer, while others were caused by lymphoma, esophageal cancer, mediastinal tumors, lung metastases, etc. Most of the 46 patients with unknown etiology (*n* = 41, 89.13%) refused pathological biopsy and diagnosis, and five patients remained unclearly diagnosed after pathological biopsy and diagnosis. As shown in Table 1, 617 BAS patients were finally enrolled and consisted of 333 (53.97%) females and 284 (46.03%) males, with an average age of 48.93 ± 18.30 (ranging from 14 to 87 years). In addition, 218 patients (35.33%) were younger than 40, 181 (29.34%) ranged from 40 to 59 years old, and 218 (35.33%) were over 60 years old. Tuberculosis (*n* = 306, 49.59%) and trauma (*n* = 179, 29.02%) were the two leading etiologies of BAS, while the other etiologies contained airway FB (*n* = 74, 11.99%), external compression (*n* = 25, 4.05%) and other etiologies (*n* = 33, 5.35%). The other etiologies covered seven cases caused by non-specific bacterial infections, five esophagotracheal fistula, three tracheobronchopathia osteochondroplastica, tracheobronchial amyloidosis and nontuberculous mycobacteriosis. They also included two bronchial calculus and bronchial hamartoma, as well as one rheumatoid arthritis with lung disease, Sjogren’s syndrome with lung disease, Rosai–Dorfman disease, tracheosquamous epithelial papilloma, trachea schwannoma, recurrent polychondritis, radiotherapy and chlorine gas (Table 2).

**Table 1:** The baseline data of benign airway stenosis cause by different etiologies.

Characteristics	Total	TBTS*	TAS^#^	Foreign body	External compression
	*n* = 617 (%)	*n* = 306 (%)	*n* = 179 (%)	*n* = 74 (%)	*n* = 25 (%)
Age at diagnosis (mean)	48.93 ± 18.30	44.57 ± 18.64	49.75 ± 16.93	58.22 ± 16.54	62.88 ± 14.04
<40	218 (35.33)	147 (48.04)	51 (28.49)	15 (20.27)	0 (0)
40–59	181 (29.34)	72 (23.53)	68 (37.99)	20 (27.03)	6 (24.00)
≥60	218 (35.33)	87 (28.43)	60 (33.52)	39 (52.70)	19 (76.00)
Sex					
Female	333 (53.97)	215 (70.26)	68 (37.99)	15 (20.27)	17 (68.00)
Male	284 (46.03)	91 (29.74)	111 (62.01)	59 (79.73)	8 (32.00)
Clinical symptoms					
Cough	483 (78.28)	281 (91.83)	100 (55.87)	67 (90.54)	8 (32.00)
Dyspnea	387 (62.72)	165 (53.92)	154 (86.03)	2 (2.70)	22 (88.00)
Fever	45 (7.29)	36 (11.76)	12 (6.70)	5 (6.76)	0 (0)
Chest pain	42 (6.81)	29 (9.48)	2 (1.12)	9 (12.16)	0 (0)
Loss of weight	14 (2.27)	14 (4.58)	6 (3.35)	0 (0)	0 (0)
Bloody sputum	16 (2.59)	12 (3.92)	2 (1.12)	2 (2.70)	0 (0)
Hemoptysis	19 (3.08)	11 (3.59)	1 (0.56)	4 (5.41)	0 (0)
Respiratory failure	20 (3.24)	4 (1.31)	10 (5.59)	0 (0)	0 (0)
Comorbidities					
Hypertension	158 (25.61)	40 (13.07)	76 (42.46)	23 (31.08)	13 (52.00)
Diabetes	78 (12.64)	18 (5.88)	37 (20.67)	9 (12.16)	9 (36.00)
Coronary heart disease	67 (10.86)	16 (5.23)	33 (18.44)	0 (0)	8 (32.00)
Cerebrovascular disease	106 (17.18)	5 (1.63)	86 (48.04)	11 (14.86)	3 (12.00)
Stenosis site					
Trachea	222 (35.98)	7 (2.29)	173 (96.65)	2 (2.70)	25 (100)
Right bronchi	203 (32.90)	147 (48.04)	4 (2.23)	46 (62.16)	0 (0)
Left bronchi	153 (24.80)	119 (38.89)	1 (0.56)	26 (35.14)	0 (0)
Multiple sites	39 (6.32)	33 (10.78)	1 (0.56)	0 (0)	0 (0)
Treatment					
Interventional therapy	512 (82.98)	259 (84.64)	168 (93.85)	72 (97.30)	2 (8.00)
Stent implantation	72 (11.67)	15 (4.90)	47 (26.26)	0 (0)	7 (28.00)
Conservative treatments	70 (11.35)	47 (15.36)	11 (6.15)	1 (1.35)	8 (32.00)
Surgery	28 (4.54)	2 (0.65)	7 (3.91)	1 (1.35)	15 (60.00)

^*^TBTS, tuberculous tracheobronchial stenosis; ^#^TAS, traumatic airway stenosis.

**Table 2:** Etiology of the 617 benign airway stenosis patients.

Characteristics	*n*	%
Tuberculosis	306	49.59%
Trauma	179	29.02%
Tracheal intubation	92	14.91%
Tracheotomy	69	11.18%
Injury	15	2.43%
Surgery	3	0.16%
Foreign body	74	11.99%
External compression	25	4.05%
Non-specific bacterial infections	7	1.13%
Esophagotracheal fistula	5	0.81%
Tracheobronchopathia osteochondroplastica	3	0.49%
Tracheobronchial amyloidosis	3	0.49%
Nontuberculoaus mycobacteriosis	3	0.49%
Bronchial calculus	2	0.32%
Bronchial hamartoma	2	0.32%
Sjogren’s syndrome with lung disease	1	0.16%
Rheumatoid arthritis with lung disease	1	0.16%
Rosai-Dorfman disease	1	0.16%
Tracheosquamous epithelial papilloma	1	0.16%
Trachea schwannoma	1	0.16%
Recurrent polychondritis	1	0.16%
Radiotherapy	1	0.16%
Chlorine gas	1	0.16%

The most common stenosis site was the trachea (*n* = 222, 35.98%), while the other stenosis sites were left (*n* = 153, 24.80%) and right bronchi (*n* = 203, 32.90%), as well as multiple sites (*n* = 39, 6.32%). In total, 512 patients (82.98%) received comprehensive interventional therapy, including balloon dilatation, high-frequency electric, laser and argon plasma thermal ablation, snare resection, etc. Furthermore, 72, 28, and 70 patients (11.67, 4.54, and 11.35%) received stent implantation, surgery and other conservative treatments, respectively.

Follow-ups were conducted on 535 of these patients by telephone, WeChat or on the site, with a period of 6 months to 5 years, while the other 82 patients dropped out. It was found that most of them (*n* = 328, 61.31%) had a partial recovery, 27.85% (*n* = 149) had a complete recovery, and 10.84% (*n* = 58) had died. Of the 58 deaths, 38 died of respiratory failure or lung infection, while the remaining 20 died of other underlying diseases (Table 3).

**Table 3:** The outcome of benign airway stenosis cause by different etiologies.

Outcome	Total	TBTS^*^	TAS^#^	Foreign body	External compression
	*n* = 535 (%)	*n* = 262 (%)	*n* = 155 (%)	*n* = 65 (%)	*n* = 24 (%)
Complete recovery	149 (27.85)	42 (16.03)	22 (14.19)	64 (98.46)	15 (62.50)
Incomplete recovery	328 (61.31)	204 (77.86)	97 (62.58)	1 (1.54)	8 (33.33)
Death	58 (10.84)	16 (6.11)	36 (23.23)	0 (0)	1 (4.17)

^*^TBTS, tuberculous tracheobronchial stenosis; ^#^TAS, traumatic airway stenosis.

### 3.2. Clinical characteristics of TBTS

The data of 306 tuberculous tracheobronchial stenosis (TBTS) patients are summarized in Table 1. A majority of patients were females (*n* = 215, 70.26%), with a mean age of 44.57 ± 18.64 (range, 14–88), including 147 (48.04%) under 40, 72 (23.53%) from 40 to 59, and 87 (28.43%) over 60. All patients developed symptoms, including cough (*n* = 281, 91.83%), dyspnea (*n* = 165, 53.92%), fever (*n* = 36, 11.76%), chest pain (*n* = 29, 9.48%), loss of weight (*n* = 14, 4.58%), bloody sputum (*n* = 12, 3.92%) and hemoptysis (*n* = 11, 3.59%). Comorbidities of TBTS in this study encompassed hypertension (*n* = 40, 13.07%), diabetes (*n* = 18, 5.88%), coronary heart disease (*n* = 16, 5.23%), cerebrovascular disease (*n* = 5, 1.63%) and respiratory failure (*n* = 4, 1.31%). TBTS primarily occurred in right bronchi (*n* = 147, 48.04%), including 70 cases in the right middle bronchus, 46 in the right main bronchi, and 31 in the right branch, followed by left bronchi (*n* = 119, 38.89%) which included 97 cases in left main bronchus and 22 in the left branch. Multiple sites (*n* = 33, 10.78%) and trachea (*n* = 7, 2.29%) were the other stenosis sites. Most TBTS patients (*n* = 259, 84.64%) were treated by interventional therapy, while 15, two and 47 received stent implantation (4.90%), surgery (0.65%), and conservative treatments (15.36%), respectively. Of the 15 patients implanted with airway stents, 10 had V-shaped silicone stents implanted, and three were implanted with Y-shaped silicone stents and two metal stents.

**Figure 1:**
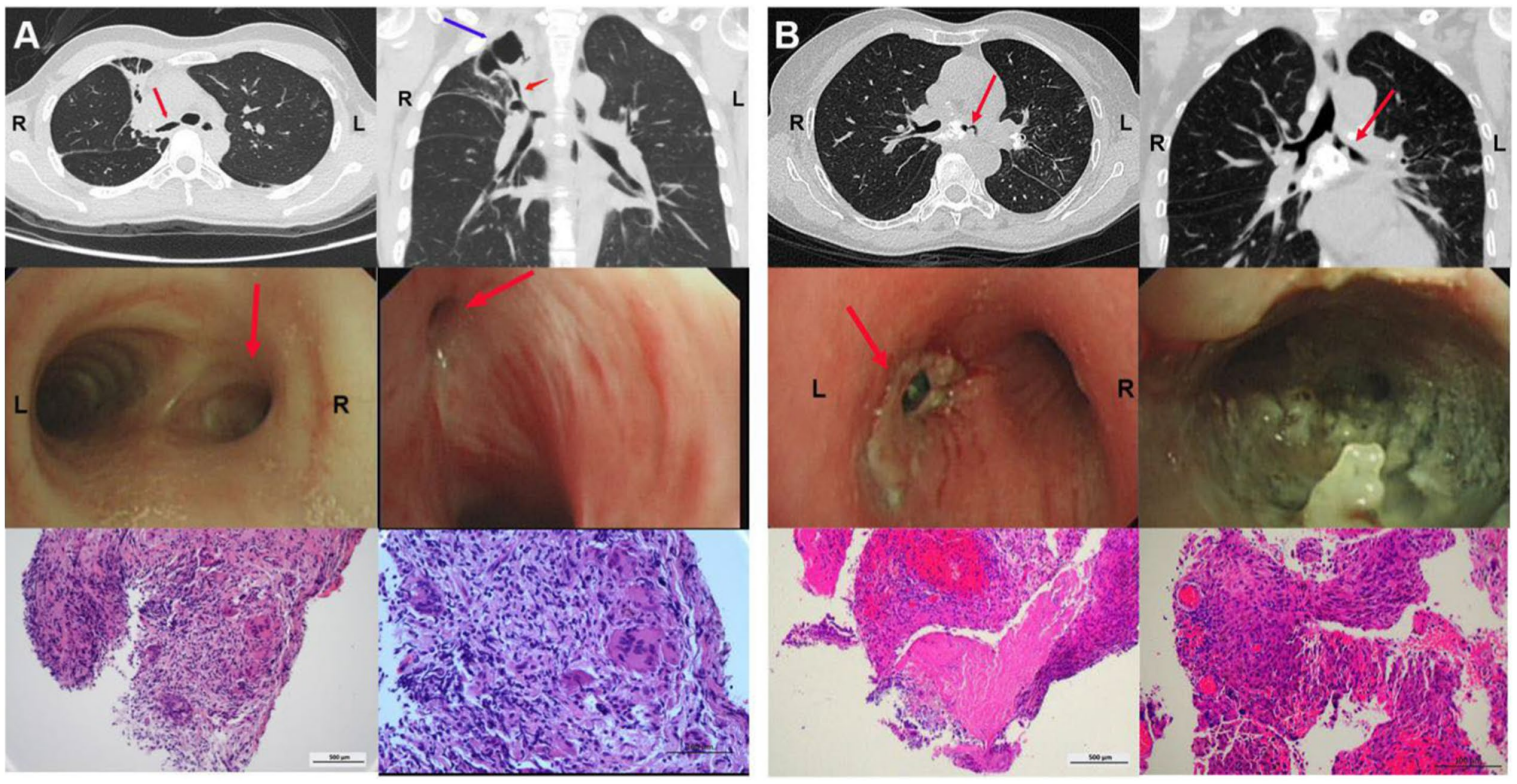
Clinical characteristics of TBTS. **(A)** CT, bronchoscopy and pathology characteristics of a 39-year-old woman diagnosed with active TBTS. A CT scan revealed stenosis in right main and right upper bronchi, and a big tuberculous cavity was found in the right upper lobe. Mucosal swelling, granulation hyperplasia, lumen deformation and stenosis were observed with a bronchoscopy. Pathology showed that extensive granulation tissue hyperplasia and acid-fast bacilli and staining were positive, with inflammatory cell aggregation. **(B)** CT, bronchoscopy and pathology characteristics of a 23-year-old woman diagnosed with active TBTS. CT scan showed stenosis in the left main bronchus and the enlargement of mediastinal lymph nodes. A bronchoscopy demonstrated the obvious stenosis of the left main bronchus, a large amount of gray and white cheesy necrosis and visible bronchial lymph node fistula. H&E staining revealed cheesy necrosis and inflammatory cell aggregation. TBTS: tuberculous tracheobronchial stenosis.

As depicted in the report of representative cases (Figure 1A), a 39-year-old female patient was diagnosed with active TBTS, whose most notable symptoms were cough and dyspnea. A computed tomography (CT) scan revealed stenosis in right main and right upper bronchi, and a big tuberculous cavity was found in the right upper lobe. Mucosal swelling, granulation hyperplasia, lumen deformation and stenosis were observed with a bronchoscopy. Pathology showed that extensive granulation tissue hyperplasia, and acid-fast bacilli and staining were positive, with the aggregation of inflammatory cells.

**Figure 2:**
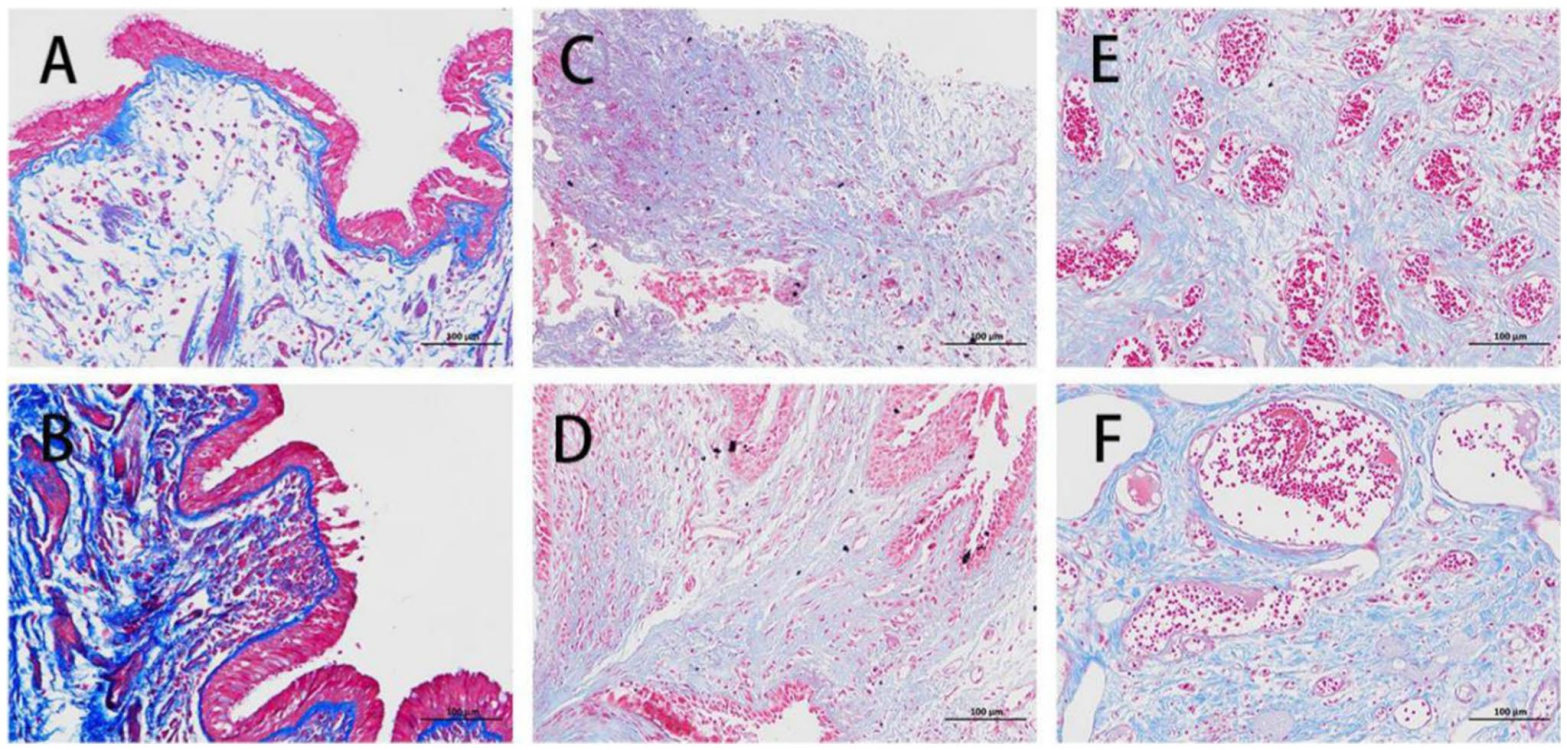
Masson’s staining of normal control and benign airway stenosis. **(A,B)** A normal control from a female patient aged 57 and diagnosed with lung cancer. **(C,D)** A male patient aged 57 and diagnosed with tuberculous tracheobronchial stenosis. **(E,F)** A male patient aged 27 and diagnosed with traumatic airway stenosis.

Another representative case report is presented in Figure 1B. In the report, a 23-year-old female patient was also diagnosed with active TBTS, whose main symptoms were cough and dyspnea. Here, the CT scan showed stenosis in the left main bronchus and the enlargement of mediastinal lymph nodes. A bronchoscopy demonstrated the obvious stenosis of the left main bronchus, a large amount of gray and white cheesy necrosis and visible bronchial lymph node fistula. H&E staining revealed cheesy necrosis and inflammatory cell aggregation. Masson staining showed fibrosis in the granulation tissues of TBTS compared with the normal control group (Figure 2).

**Figure 3:**
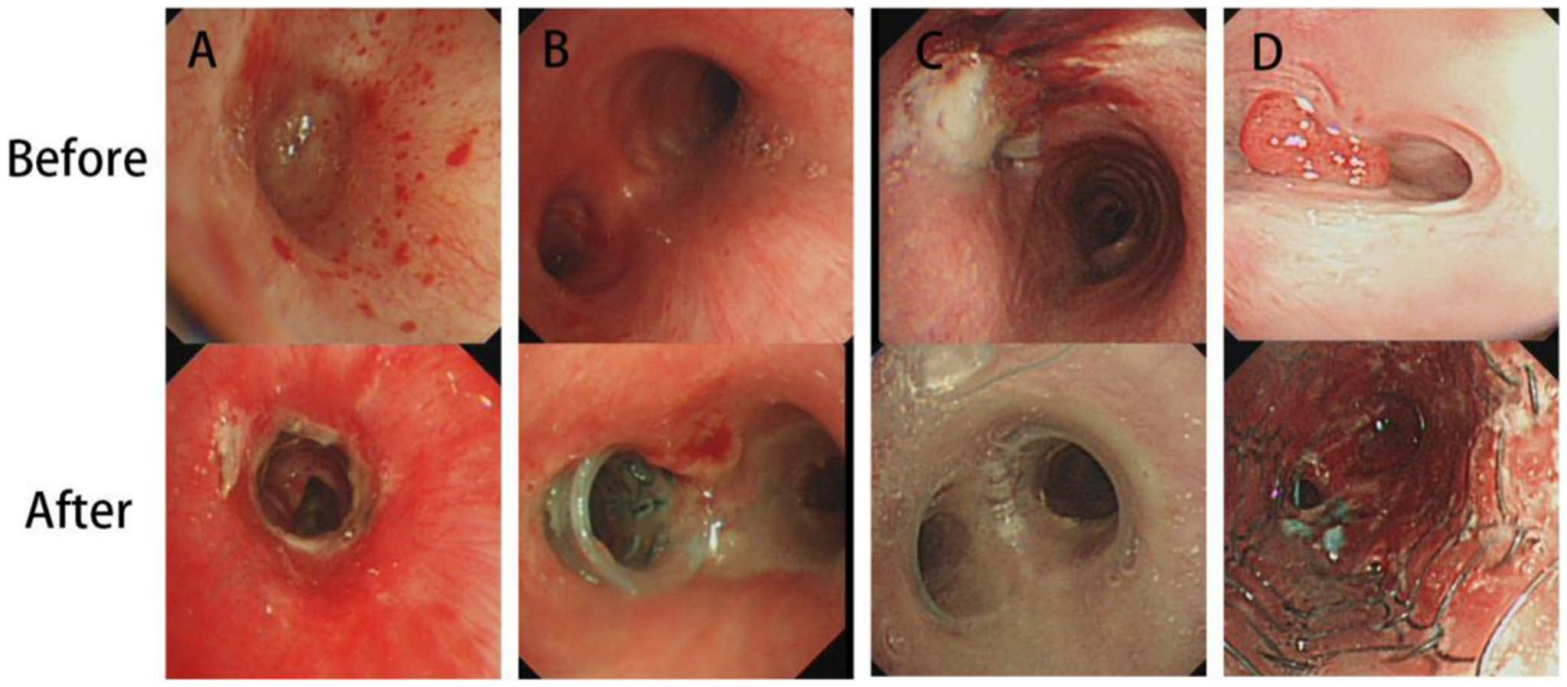
Treatment methods of tuberculous tracheobronchial stenosis. **(A)** Interventional therapy. **(B)** V-shape silicone stent. **(C)** Y-shape silicone stent. **(D)** Metallic stent.

Here, different treatments are shown in Figure 3. A was a patient treated by interventional therapy, while B, C and D were implanted with V-shape silicone, Y-shape and metallic stents, respectively. Airway stenosis was relieved after interventional therapy or stent plantation. Of 262 TBTS patients followed up, 42 (16.03%) had a complete recovery after treatment, 204 (77.86%) had an incomplete recovery, and 16 (6.11%) died of respiratory failure or lung infection (Table 3).

### 3.3. Clinical characteristics of traumatic airway stenosis

A total of 179 traumatic airway stenosis (TAS) patients were enrolled in this study (Table 1). Their etiologies included tracheal intubation (*n* = 92), tracheotomy (*n* = 69), injury (*n* = 15), and surgery (*n* = 3), which mainly occurred in the trachea (*n* = 173, 96.65%). Four cases (2.23%) were located in right bronchi, one (0.56%) in left bronchi, and one in multiple sites. Most of the TAS patients were males (*n* = 111, 62.01%), with a mean age of 49.75 ± 16.93 (range 14–82). A total of 51 patients (28.49%) were younger than 40, 68 (37.99%) were aged between 40 and 59, and 60 (33.52%) were at the age of over 60. Their main symptoms were dyspnea (*n* = 154, 86.03%) and cough (*n* = 100, 55.87%). Nearly half of them were complicated with cerebrovascular disease (*n* = 86, 48.04%), hypertension (*n* = 76, 42.46%) and diabetes (*n* = 37, 20.67%).

**Figure 4:**
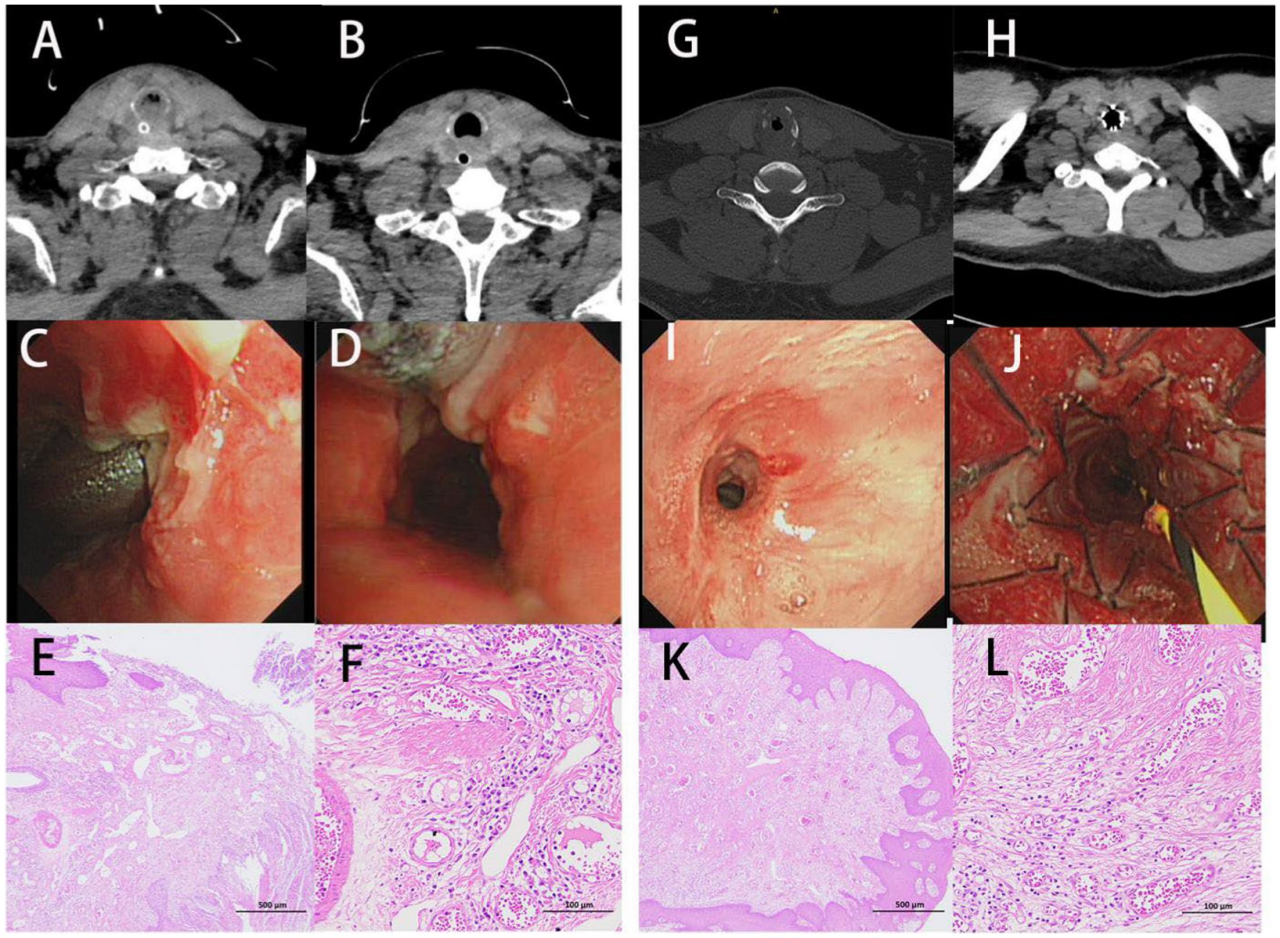
Clinical characteristics of traumatic airway stenosis patients. **(A–F)** A man aged 51 and diagnosed with post-tracheotomy stenosis. **(G–L)** A man aged 27 and diagnosed with post-intubation stenosis. **(A,C,G,I)** CT and bronchoscopy before treatment. **(B,D,H,J)** CT and bronchoscopy after treatment. **(E,F,K,L)** H&E staining.

As depicted in the report of representative cases (Figure 4), a 51-year-old man was diagnosed with post-tracheotomy stenosis and a 27-year-old man with post-intubation stenosis. Their most notable symptoms were dyspnea. CT and bronchoscopy showed severe tracheal stenosis, which resulted from massive granulation tissue hyperplasia. Pathology showed extensive granulation tissue hyperplasia. The staining results revealed the presence of squamous epithelial hyperplasia, the severe thickening of mucosa, hyperplasia, the repair of scar tissues in lamina propria and submucosa, the staggered formation of numerous mature and coarse collagen fibers, the appearance of countless capillaries, frequent vascular dilatation, mild inflammatory reaction, and the infiltration of a few lymphocytes. Masson’s staining indicated the proliferation of plentiful blue collagen fibers (Figure 2). All of them received interventional therapy, and stenosis was relieved.

Among 179 patients, 168 (93.85%) received comprehensive interventional therapy, including 47 (26.26%) cases who received stent implantation (36, eight, two and one cases of straight silicone stent, Montgomery T-tube implantation, metal stent and Y-shaped silicone stent, respectively), and seven (3.91%) who received surgical treatment. Finally, 11 cases (6.15%) received conservative medical treatment. Of 155 TAS patients followed up, 22 (14.19%) had a complete recovery after treatment, 97 (62.58%) had an incomplete recovery, and 16 and 20 (10.32 and 12.91%) died of respiratory failure or lung infection and other underlying diseases, respectively (Table 3).

### 3.4. BAS caused by airway FB

A total of 74 cases of airway stenosis caused by airway FB were reported, including 59 males (79.73%) and 15 females (20.27%), with an average age of 58.22 ± 16.54 (range 14–87). Thirty-nine cases (52.70%) were aged over 60, and 15 (20.27%) were younger than 40. Most of the patients appeared to have a cough (*n* = 67, 90.54%), while some seemed to experience chest pain (*n* = 9, 12.16%), fever (*n* = 5, 6.76%), hemoptysis (*n* = 4, 5.41%), dyspnea (*n* = 2, 2.70%) and hemoptysis (*n* = 2, 2.70%). A history of definite choking was present in 52 (70.27%) patients. The longest case was a 28-year-old man who inhaled a whistle 18 years ago. Twenty-three cases (31.08%) were combined with hypertension, and 11 (14.86%) with cerebrovascular disease. Most airway FBs (*n* = 46, 62.16%) were in the right bronchus, while 26 (35.14%) were in the left bronchus, and two were in the trachea (Table 1).

**Figure 5:**
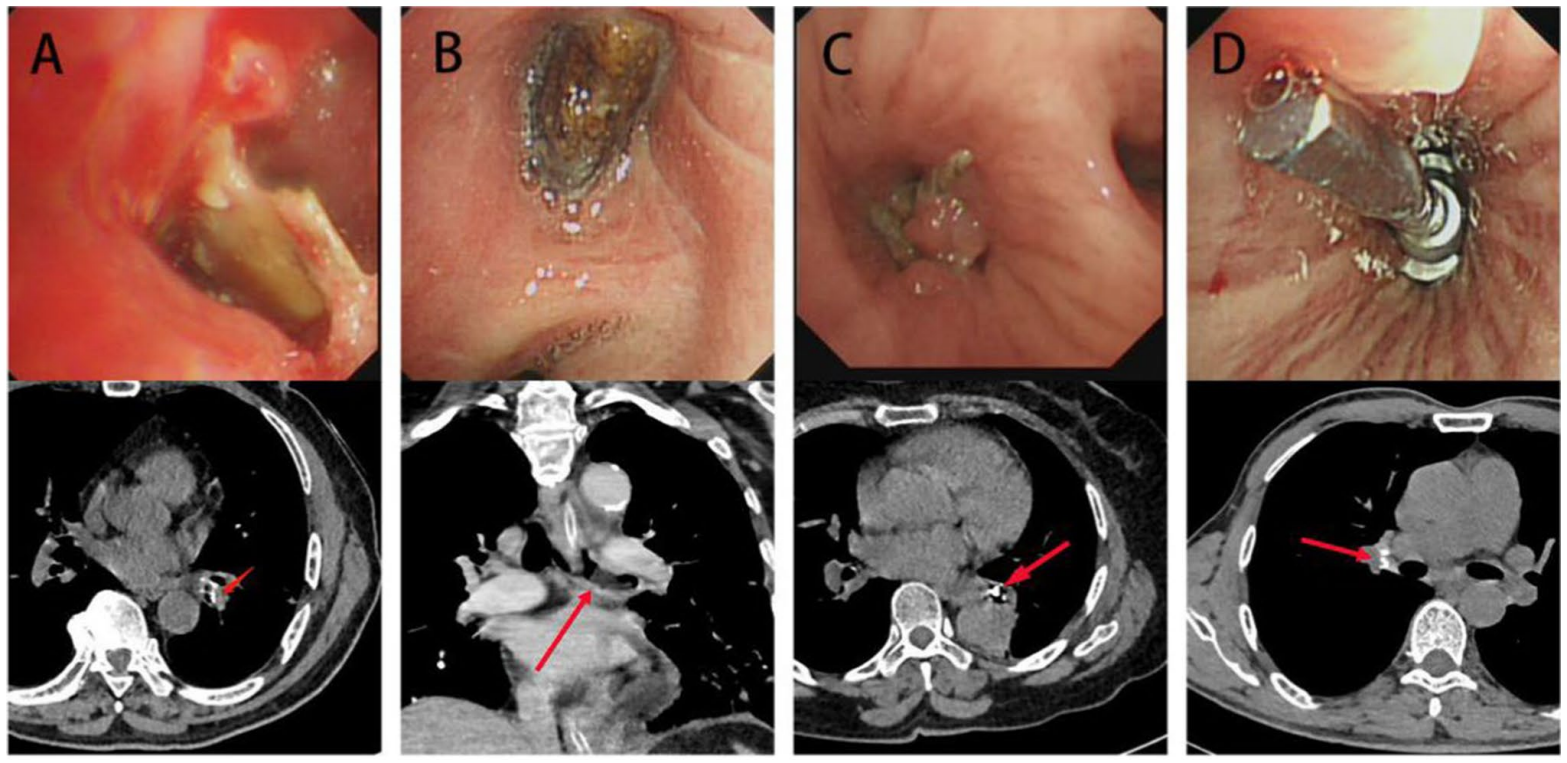
CT and bronchoscopy characteristics of different airway FBs. **(A)** A bone-like FB in the right inferior lobar bronchus. **(B)** A walnut in the left main bronchus. **(C)** A false tooth in the right inferior lobar bronchus. **(D)** A metallic FB^*^ in the left inferior lobar bronchus. FB, foreign body.

The most common airway FBs included bones (*n* = 42, 56.76%), followed by nuts (*n* = 6), false teeth (*n* = 3), capsule drug (*n* = 1), and metallic (*n* = 5, caused by dental operation), plastic (*n* = 3), and unknown FBs (*n* = 14), as mentioned in Table 4. Among them, 59 cases (79.73%) had surrounding granulation tissue formation that led to airway stenosis, and 15 (20.27%) had no granulation tissue formation. Most of them (*n* = 72, 97.30%) received interventional comprehensive treatment, while only one received surgical treatment because of hemoptysis caused by FB. Different types of airway FBs are presented in Figure 5. Of the 65 patients we followed, none died, most patient was complete recovery, and only one case was not completely removed the FB.

**Table 4:** Different types of airway foreign bodies (*n* = 74).

Types	*n*	%
Bones	42	56.76%
Unknown	14	18.92%
Nuts	6	8.11%
Metal foreign bodies	5	6.76%
False teeth	3	4.05%
Plastic foreign bodies	3	5.41%
Capsule drug	1	1.35%

### 3.5. BAS caused by external compression

A total of 25 BAS cases were attributable to the external compression of surrounding airway tissues (Table 1), including eight males (32.00%) and 17 females (68.00%), with an average age of 62.88 ± 14.04 (range 31–83). Dyspnea was observed to be the main symptom (*n* = 22, 88.00%), and cough was found in eight cases (32.00%). All 25 cases of stenosis were located in the main trachea, of which 22 (88.00%) were caused by thyroid goiter compression and the other three by mediastinal cyst, bronchial cyst and cervical hematoma compression, respectively. Seven patients received metal stent plantation. Two, 15 and eight patients received interventional therapy, surgical treatment and conservative treatment, respectively. The tracheal mucosa was intact, and the tracheal segment was compressed and deformed under bronchoscopy. Tracheal stenosis can be relieved after surgical treatment in 15 patients. Of the 24 patients we followed, only one died of heart disease.

**Figure 6:**
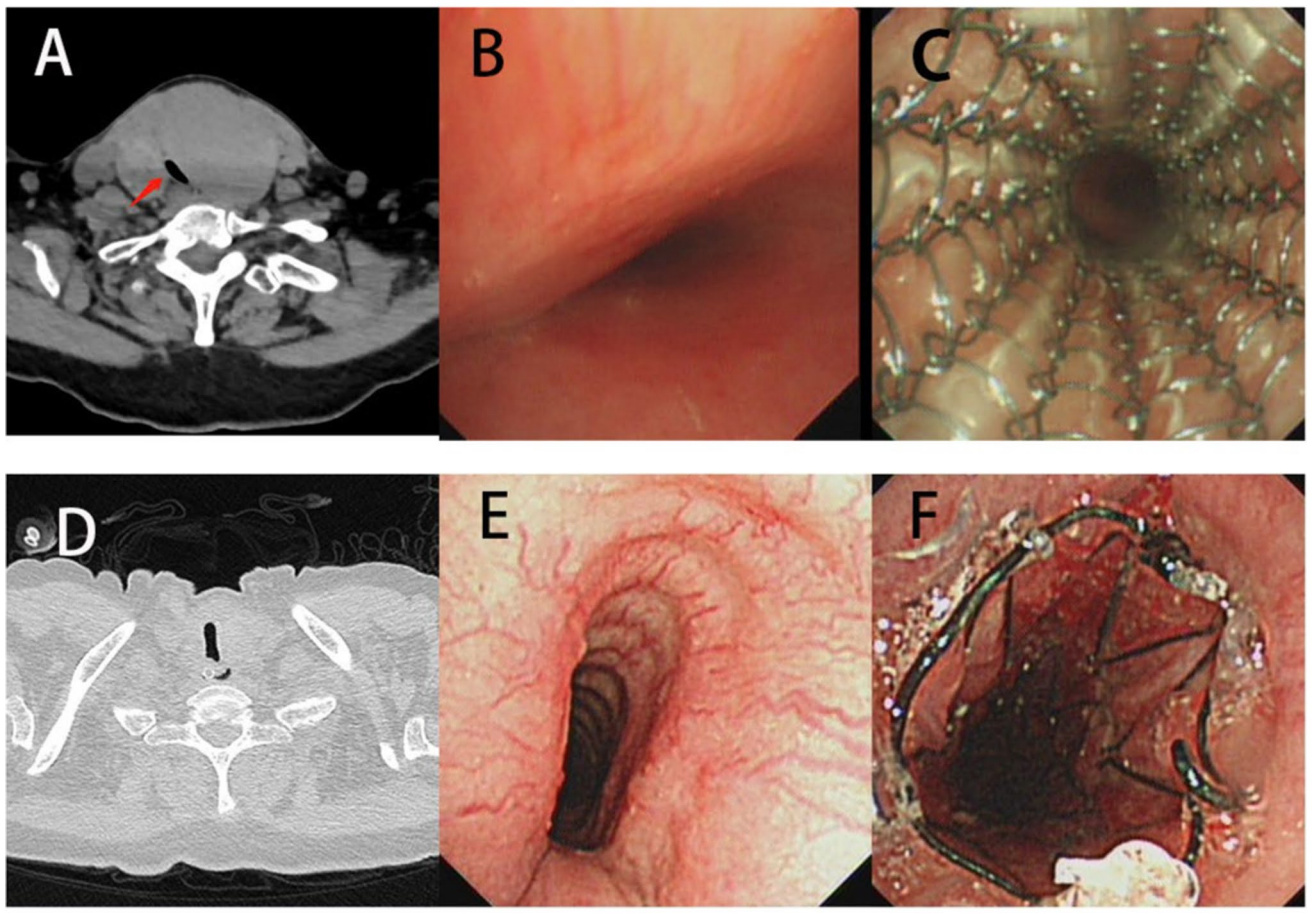
Benign airway stenosis caused by external compression. **(A-C)** CT and bronchoscopy characteristics of a 58-year-old woman, airway narrow was relieved after the plantation of a metallic stent. **(D-F)** CT and bronchoscopy characteristics of a 64-year-old woman, airway narrow was relieved after the plantation of a metallic stent.

As depicted in the report of representative cases (Figure 6), a 58-year-old woman was admitted to hospital due to “cervical mass found for more than one decade and shortness of breath for 2 days.” CT showed huge cervical goiter, and the upper tracheal segment was compressed. Bronchoscopy showed severe external compression stenosis in the trachea, and the tracheal mucous was normal. The tracheal stenosis was improved significantly after the implantation of a metal-coated stent.

Another representative case showed a 64-year-old woman admitted to hospital due to “shortness of breath for more than 1 month” and confirmed to have external compression tracheal stenosis by CT and bronchoscopy. The tracheal stenosis was also improved significantly after the implantation of a metal-coated stent (Figure 6).

### 3.6. IHC

**Figure 7:**
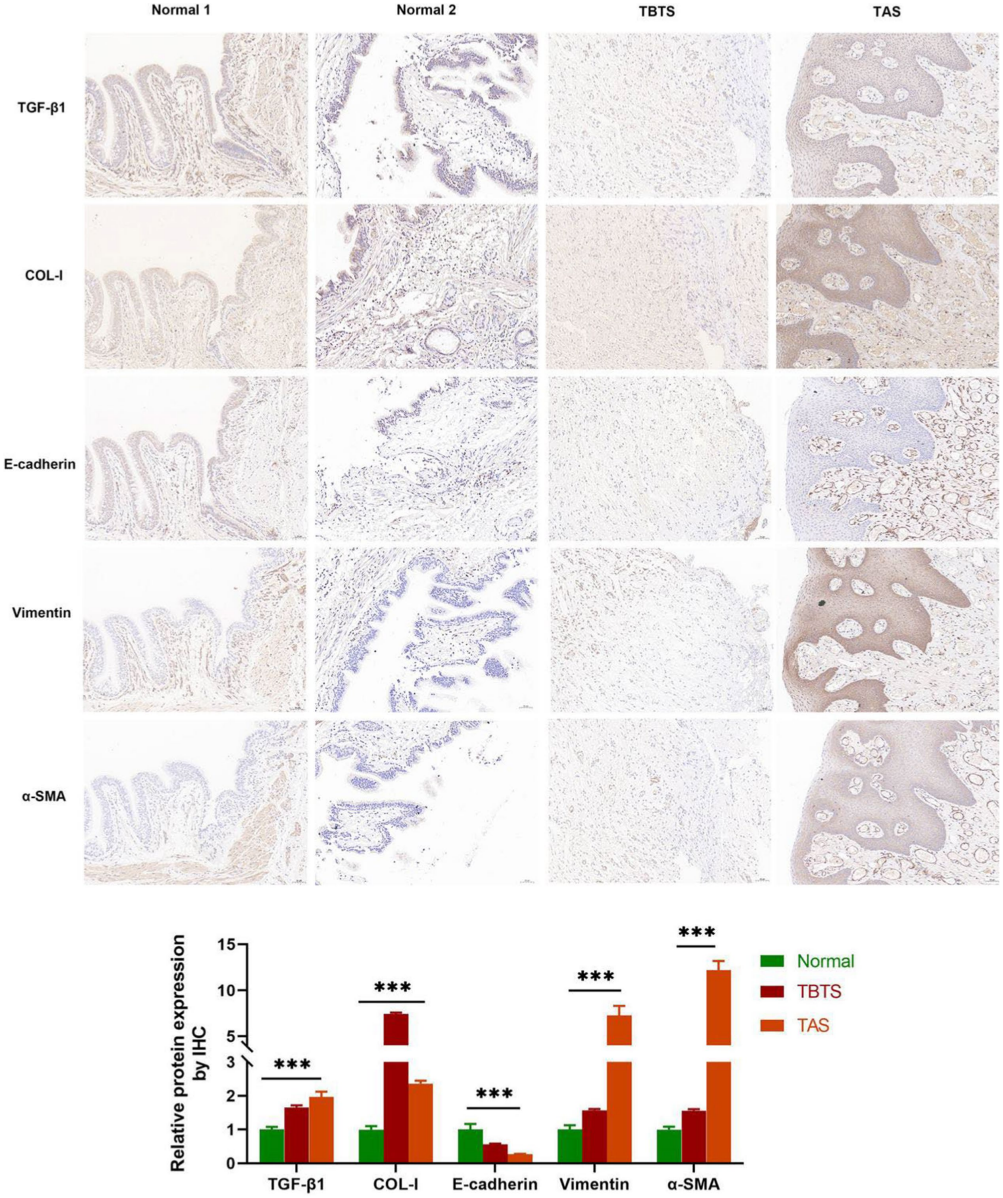
IHC of benign airway stenosis granulations and normal control tissues (20X). Normal 1: A normal control from a female patient aged 57 and diagnosed with lung cancer. Normal 2: A normal control from a male patient aged 49 and diagnosed with lung cancer. TBTS: A male patient aged 57 and diagnosed with tuberculous tracheobronchial stenosis. TAS: A male patient aged 27 and diagnosed with traumatic airway stenosis. ***P < 0.001.

IHC detection was performed on BAS granulation and normal tissues. Normal tissues were collected from a 57-year-old female patient and a 49-year-old male patient who accepted pulmonary lobectomy on account of lung adenocarcinoma and were diagnosed with lung cancer. BAS granulation tissues were gathered from a 57-year-old male patient diagnosed with TBTS and a 27-year-old male patient diagnosed with TAS. As evidenced in Figure 7, the expression of E-cadherin in BAS granulation tissues was down-regulated compared with normal control, while those of vimentin, α-SMA, COL-I, and TGF-β1 were up-regulated, with statistical significance (*p* < 0.001). These results suggested that EMT, fibrosis and the increased expression of TGF-β1 existed in BAS granulation tissues.

## 4. Discussion

In the present study, the most common cause of BAS was found to be tuberculosis, and the other causes included trauma, FB and external compression. This finding was consistent with other research in China (5, 6), which was possibly ascribed to the higher incidence of tuberculosis in this country (7, 8). China has a high incidence of tuberculosis owing to its underdeveloped economy and poor medical conditions. However, the etiology of BAS reported abroad differs from that reported in China (9). For instance, the main cause of 40 BAS patients reported by the University of California was lung transplantation (32.5%), followed by tracheotomy or tracheal intubation (25.0%) (10). Follow-ups were conducted on 535 of these patients in 5 years. It was found that most of them had partial recovery, while some died of respiratory failure, lung infection or other underlying diseases. It was shown that BAS was a chronic disease with low cure and death rates.

TBTS mainly occurred in the left main bronchus and the right middle bronchus, which could be put down to the anatomical features in these sites (2). It was less common in multiple sites or the trachea. Its common symptoms included cough and dyspnea, and some patients appeared to have fever and loss of weight. Most TBTS patients were females, whose mean age was less than TAS patients. This was in line with the other research, which may imply that TBTS was related to estrogens (11). Typical pathological characteristics like cheesy necrosis and acid-fast bacillus were noted in TBTS granulation tissues, but fibrosis and collagen hyperplasia mainly appeared in post-tuberculous cicatricial airway stenosis tissues (12). Most of the patients had received comprehensive interventional therapy, including balloon dilatation, high-frequency electric, laser and argon plasma thermal ablation, snare resection, etc. due to less trauma and low cost (2, 5). Some TBTS patients received stent implantation, including V-shaped silicone, Y-shaped silicone and metal stents. Y-shaped silicone and metal stents are widely used in BAS by virtue of low mobility (13). V-shaped silicone stents are a new type of stents with the advantages of low mobility, easy removal and few complications (14).

TAS was caused mainly by tracheal intubation and tracheotomy in this and other research, and sometimes by surgery or injury (15). The majority of TAS patients were complicated with cerebrovascular disease, hypertension and diabetes in that they were more likely to require long-term intubation or tracheotomy. Typical pathological characteristics included fibrosis in TAS granulation tissues. H&E and Masson’s staining indicated that TAS granulation tissues were mostly at the granulation hyperplasia or cicatricial stage, with a light inflammatory reaction, while fibrosis remained dominant in BAS, consistent with other studies (16, 17). Most of the TAS patients chose comprehensive interventional therapy, some chose stent plantation, and a small number of them chose surgery. The choice between interventional therapy, surgery or stent plantation in TAS is individualized according to stenosis site and length. Interventional therapy would be selected for TAS patients with mild lesions, and stent implantation would be selected for those with severe lesions, tracheal cartilage collapse or asphyxia risk. Additionally, surgical treatment would be selected for TAS patients with extensive lesions or ineffective stent treatment (18). Stent implantation included straight silicone, Y-shape silicone, Montgomery T-tube and metallic stents. Montgomery T-tube implantation is a safe, viable and effective tracheal forming method for airway stenosis caused by tracheotomy (19).

In the present study, organic FBs were shown to be the most common airway FBs that resulted in BAS, including bones, nuts and other food. Most of the patients had a history of definite choking (20). Iatrogenic FBs are also common in adults’ airway FBs, almost caused by dental operation (21). The most common site was the right bronchus because of its anatomical characteristics, which were aligned with other studies (22, 23). Timely and effective interventional therapy is an important way to prevent BAS caused by airway FB (23).

In general, BAS caused by external compression is triggered by thyroid goiter, lymphoma, granuloma, esophageal tumor or large vascular malformation, etc. The airway mucosa is mostly normal, and only the compression of surrounding tracheal tissues gives rise to the occurrence of lumen stenosis and cartilage deformation (24, 25). For BAS patients caused by thyroid goiter, most were relieved by surgery, while some may have comorbidities and require a multidisciplinary approach to management owing to complications like tracheomalacia or respiratory failure after surgery (24). The other etiologies included benign airway tumor, congenital airway stenosis, oxygen inhaling and tracheobronchomalacia (26, 27).

IHC analysis revealed that the expression of E-cadherin in BAS granulation tissues was down-regulated, while those of vimentin, α-SMA, COL-I, and TGF-β1 were up-regulated compared with the normal control group. This suggested that EMT, fibrosis and the increased expression of TGF-β1 existed in BAS granulation tissues, which accorded with other studies (12, 28, 29). TGF-β1 is of importance to the occurrence and development of BAS and is activated under stress like inflammation and injury. It promotes the occurrence and development of EMT, fibroblast-to-myofibroblast transformation, and fibrosis through various pathways. In addition, TGF-β1 facilitates the mesenchymal transformation of epithelial cells, the differentiation of fibroblasts into myofibroblasts, and the proliferation and migration of myofibroblasts (30). Some research has demonstrated that EMT appears in BAS, usually at the early stage of the disease (12, 28).

In this study, tuberculosis was the most common cause of BAS. Therefore, the prevention and timely treatment of tuberculosis became critical to preventing BAS. Likewise, managing patients with tracheal intubation and tracheotomy is also a top priority in BAS prevention.

## 5. Conclusion

The clinical characteristics of 617 BAS patients caused by different etiologies were analyzed and summarized. Tuberculosis was the main etiology, and trauma was the secondary etiology. The granulation tissues of BAS were characterized by inflammation, fibrosis and probably EMT. Comprehensive interventional therapy is an effective method of treating BAS, while airway stent plantation and surgery are used to supplement those without responding to conventional interventional therapy. This study is limited because it only includes cases from one hospital. It may contribute to the development of more personalized approaches to the diagnosis and management of BAS in the future.

## Data availability statement

The raw data supporting the conclusions of this article will be made available by the authors, without undue reservation.

## Ethics statement

The study was approved by the Ethics Committee of the Second Affiliated Hospital of Guangxi Medical University, Nanning, China. Approval number [No.2021-KY(0172)] and was conducted according to the principles outlined in the Declaration of Helsinki. Informed consent was obtained from all the subjects. Written informed consent to participate in this study was provided by the participants' legal guardian/next of kin.

## Author contributions

JW and SQ: conceptualization and writing-review and editing. WL, YC, and TF: collecting samples and experiment implementation. ST and YW: collecting and analyzing data. GL: supervision and funding acquisition. All authors read and agreed to the published version of the manuscript.

## Funding

This work was supported by the Guangxi Clinical Medical Research Center for Respiratory Diseases (No. 2022AC04005), the National Nature Science Foundation of China (No. 82060003), and the Self-funded Project of Guangxi Health Commission (No. Z20211021).

## Conflict of interest

The authors declare that the research was conducted in the absence of any commercial or financial relationships that could be construed as a potential conflict of interest.

## Publisher’s note

All claims expressed in this article are solely those of the authors and do not necessarily represent those of their affiliated organizations, or those of the publisher, the editors and the reviewers. Any product that may be evaluated in this article, or claim that may be made by its manufacturer, is not guaranteed or endorsed by the publisher.
